# Application of an Empirical Extreme Value Distribution to Load Models

**DOI:** 10.6028/jres.099.039

**Published:** 1994

**Authors:** Jun Kanda

**Affiliations:** University of Tokyo, Tokyo 113, Japan

**Keywords:** annual maximum, equivalent uniformly distributed load, extreme live load, extreme value distribution, Frechet distribution, Gumbel distribution, maximum bedrock velocity, upper bound value, windspeed

## Abstract

An empirical extreme value distribution with lower and upper bounds proposed by the author is applied to represent probability distribution models for maximum load intensities of (he earthquake ground motion, the wind speed, and the live load in supermarkets. One of the difficulties in the estimation of the parameters is determining the upper bound value. Nevertheless application of the proposed distribution to the annual maximum earthquake ground motion results in considerable improvements over other models. Possible improvements to the annual maximum wind speed model are discussed. The proposed distribution is also a good candidate for the live load extremes.

## 1. Introduction

Probabilistic load models are utilized for limit state design procedures and safety assessments of structures. Since lifetime maximum loads have to be applied to these analyses, appropriate probability distributions are needed to represent load intensity models. The Gumbel distribution (Type I extreme value) and the Frechet distribution (Type 11 extreme value) are often used for such purposes.

When the coefficient of variation (cov) is not large, discrepancies of the upper tails may not be very serious. However for load intensities with fairly large cov such as earthquake ground motions, existing extreme value distributions do not provide good fits to statistical data. Then an empirical extreme value distribution with both upper and lower bounds proposed by the author [1] is a good alter- native to improve probabilistic load models.

Statistical data were prepared for the annual maximum earthquake ground motion, the annual maximum wind speed, and live load extremes in- crowding situations. The significance of the proposed distribution is discussed in terms of lifetime maximum statistics.

## 2. Proposed Extreme Value Distribution

Three types of extreme value distributions are commonly used for engineering purposes. Cumulative distribution functions are written as follows [2],

$$F_{1}=\exp[-\exp\{-a(x-b)\}]\;-\infty <x<\infty$$ (1)

$$F_{11}(x)=\exp\left[-{\left(\frac{c}{x-\in }\right)}^{\gamma }\right]\;\in <x<\infty$$ (2)

$$F_{111}(x)=\exp\left[-{\left(\frac{w-x}{w-\upsilon }\right)}^{\gamma }\right]\;-\infty <x<w$$ (3)

where *a, b, c, ϵ, γ, w, υ* are parameters which characterize the form of distribution. Distributions expressed in Eqs. (1), (2), and (3) are the Gumbel, Frechet and Weibull distributions respectively. In Eq. (1), the random variable *x* could theoretically vary between −∞ and *+* ∞, while in Eq. (2) the lower bound value, ϵ, and in Eq. (3) the upper bound value, w, exist. When natural phenomena are considered, it seems reasonable that the physical quantity has a positive value with an upper bound limit. On consideration the formula of Eqs. (2) and (3), the following empirical extreme value distribution has been proposed [1]

$$F_{J}(x)=\exp\left[-{\left\{\frac{w-x}{u(x-\epsilon }\right\}}^{\gamma }\right],\;\epsilon <x<\epsilon$$ (4)

where *w* and *ϵ* are upper and lower bound values, respectively, and *u* and *γ* are scale and shape parameters, respectively. It can be seen that when *x* approaches the lower bound, *ϵ*, Eq. (4) approaches Eq. (2) with *c =(w− ϵ*)/*u* and when *x* approaches the upper bound, *w*, Eq. (4) approaches Eq. (3) with *v* = *w* −*u*(*w* − ϵ).

**Fig. 1:**
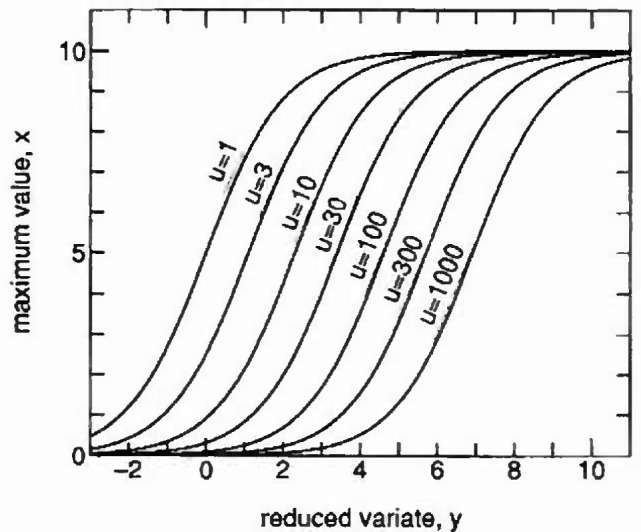
Effect of parameter u on proposed distribution with *w* = 10, *γ* =1.0. (Gumbel distribution probability paper.)

**Fig. 2:**
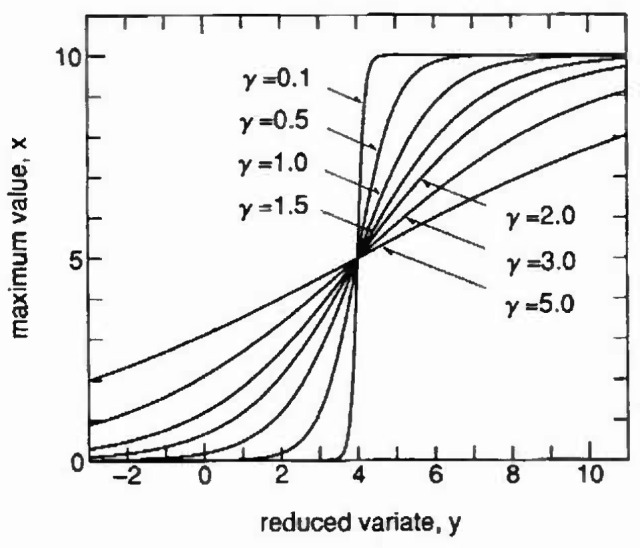
Effect of parameter *y* on proposed distribution with *w* = 10, *u* = exp(4/*γ*). (Gumbel distribution probability paper.)

In order to demonstrate the form of the proposed distribution, the effect of parameter *u* with *w* = 10 and *γ* = 1.0 for Eq. (4) is shown in Fig. 1 on Gumbel probability paper, with *x* on the ordinate and reduced variate *y* on the abscissa. In a similar way the effect of parameter *γ* with *w* = 10 and *u* = exp(4/*γ*) for Eq. (4) is demonstrated in Fig. 2.

## 3. Annual Maximum Earthquake Ground Motion Model

The seismic hazard estimation has often been based on earthquake occurrence models assuming a Gutenberg-Richter relationship. An alternative estimation is possible when sufficient number of earthquake records are available to acquire annual maximum earthquake ground motion data at a site. Such an approach is rather common in Japan beginning with Kawasumi’s work in 1951 [3]. A recent attempt was made by applying the proposed distribution of Eq. (4) [4]. Some modifications were introduced in this study. Earthquake data for the last 400 years were utilized according to Usami’s catalogue [5]. Kanai’s attenuation law was chosen as a representative relationship between the bedrock velocity, *V*, and the magnitude, *M*, with the hypocentral distance, *x*, expressed in the following formula [6]

$$V=10^{0.6LM-(1.66+\frac{36}{x})\;\log\;x-(0.631+\frac{183}{x})}$$ (5)

where a focal depth of 30 km is uniformly assumed to calculate *x*.

**Fig. 3:**
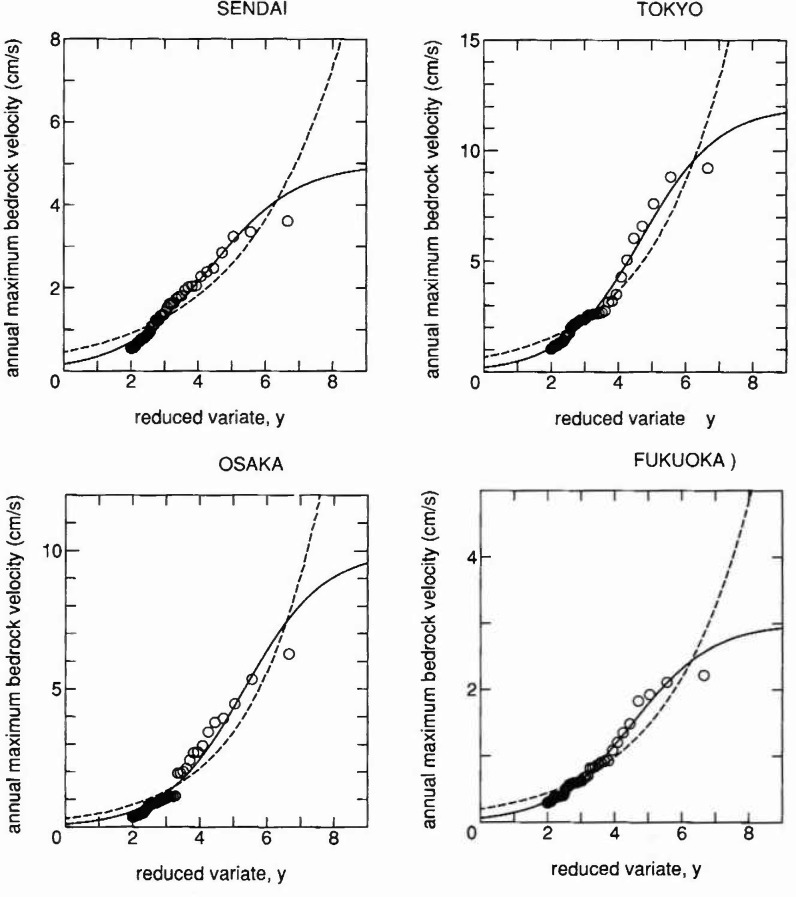
Extreme value fitting to annual maximum bedrock velocity of earthquake motion in Japan, where the solid and dashed lines indicate the proposed and the Frechet distribution, respectively.

The annual maxima of bedrock velocity calculated according to Eq. (5) are plotted on Gumbel probability paper for four sites, i.e., Sendai, Tokyo, Osaka, and Fukuoka in Fig. 3. The 50 largest data from annual maxima in the 400 year period were used, since minor earthquake motions were considered to be missing in historical records or documents and so should be eliminated from the analysis.

The upper bound value could be assumed from tektonic findings on the fault activity [4], however the upper bound magnitude seems to provide a fairly rough estimate for a particular site as a variation of magnitude by 0.1 causes only 15% change in the estimation of *V*.

The proposed distribution fitted to plotted data by the least squares method is shown where the value of *w* in Eq. (4) was chosen by engineering judgment as *w* = 5.0, 12.0, 10.0 and 3.0 for Sendai, Tokyo, Osaka and Fukuoka, respectively. The Frechet distribution fitted by the same method is also shown in a dashed line for comparison. The existence of saturation tendency indicates that the representation by Eq. (4) is better.

The difference in the results between the proposed distribution and the Frechet distribution can be summarized in Table 1. The error is estimated in terms of the normalized square root of sum of squares error as,

$$E=\sqrt{\frac{1}{n}\sum {\left(x_{i}-{\hat{x}}_{i}\right)}^{2}}/\frac{1}{n}\sum x_{i},$$ (6)

where *x_i_* is the *i* th annual maximum data andx.is the corresponding value estimated from the distribution model, and n = 50 for earthquake models.

Although the extimated mean of 50 year maximum values based on the Frechet distribution is similar to that based on the proposed distribution, estimated cov values for the 50 year maxima based on the Frechet distribution, which are the same as those for annual maxima, are considerably greater than those yielded by the proposed distribution. Significant reduction in the error estimate also indicates the appropriateness of the proposed distribution. The cumulative distribution of the 50 year maximum was obtained as the 50th power of the cumulative distribution function of the annual maximum, i.e., it was assumed that the annual maxima are mutually independent. The mean and cov of the 50 year maximum were calculated numerically for the proposed distribution as the closed form relationship between the mean and variance and the parameters in Eq. (4) is not obtainable.

## 4. Annual Maximum Wind Speed

The Gumbel distribution is often used to represent the annual maximum wind speed distribution. The possibility of improved representation by Eq. (4) is examined for sites where some saturation tendencies are observed, i.e., Aomori, Akita, Nagoya and Kagoshima.

**Fig. 4:**
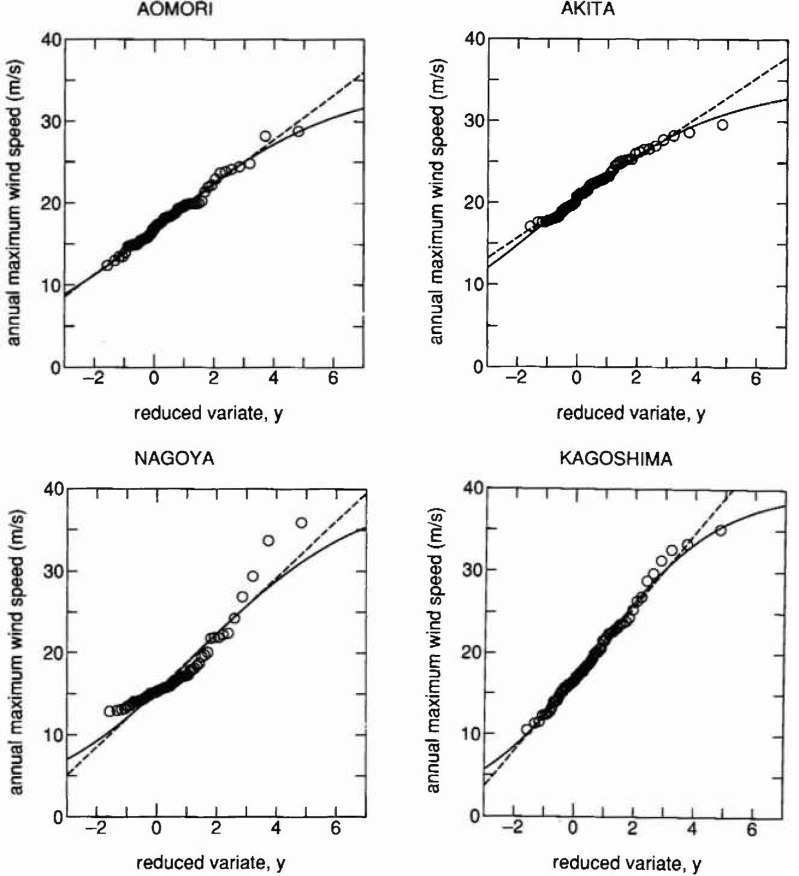
Extreme value fitting to annual maximum wind speed in Japan, where the solid and dashed lines indicate the proposed and the Gumbel distribution, respectively.

The wind speed data were corrected by taking into account changes of measurement height and the change of the terrain roughness in the period between 1960 and 1970 [7]. Measured data at meteorological agency stations in the period between 1929 and 1991 were utilized. Plotted data and distribution curves fitted to the plots, as was done for the earthquake cases, are shown in Fig. 4, with dashed lines representing the Gumbel distribution. The upper bound *w* = 35 (m/s) was used for Aomori and Akita, while *w* =40 was used for Nagoya and Kagoshima in Eq. (4). Although the difference between the two types of distributions is not as significant as in the case of earthquakes, error estimates arc improved except for Nagoya, where the fitting is rather poor in comparison with other cases as seen in Table 2. The use of a nonzero lower bound value, e.g., *ϵ* = 10, could improve the fitting for the case of Nagoya. However, this was avoided. Two different major factors, such as the occurrence of typhoons and monsoons, could be the reason for the concave shape of plots on the Gumbel probability paper.

Estimated mean and cov values for the annual maxima and the 50 year maxima are also listed in Table 2. The reduction in estimation of cov of the 50 year maximum for the proposed distribution can be pointed out. When the existence of an upper bound for the extreme value distribution of wind speed is accepted, such a reduction could result in a smaller load factor in the probability-based design.

## 5. Simulated Extreme Live Load

Extraordinary live loads for the ultimate limit state design may be estimated based on computer simulations according to a scenario for extraordinary situations. One reported possibility is to model crowd-gathering situations in supermarkets [8]. Room plans with specified shapes and weights of racks with goods and other furnishings were used according to surveyed data, and crowd-gathering situations at one corner of each room were simulated for typical cases such as,

- from 0.3 person/m^2^ to 5.0 person/m^2^
- from 1.0 person/m^2^ to 10.0 person/m^2^

Personnel loads were distributed in the area with no racks or furniture, and 700 *N* was postulated as the weight of a person.

The equivalent uniformly distributed loads (EUDL) were calculated for slab end bending moments in a shorter span and for girder end bending moments. The detailed procedure is described else- where [8].

**Fig.5:**
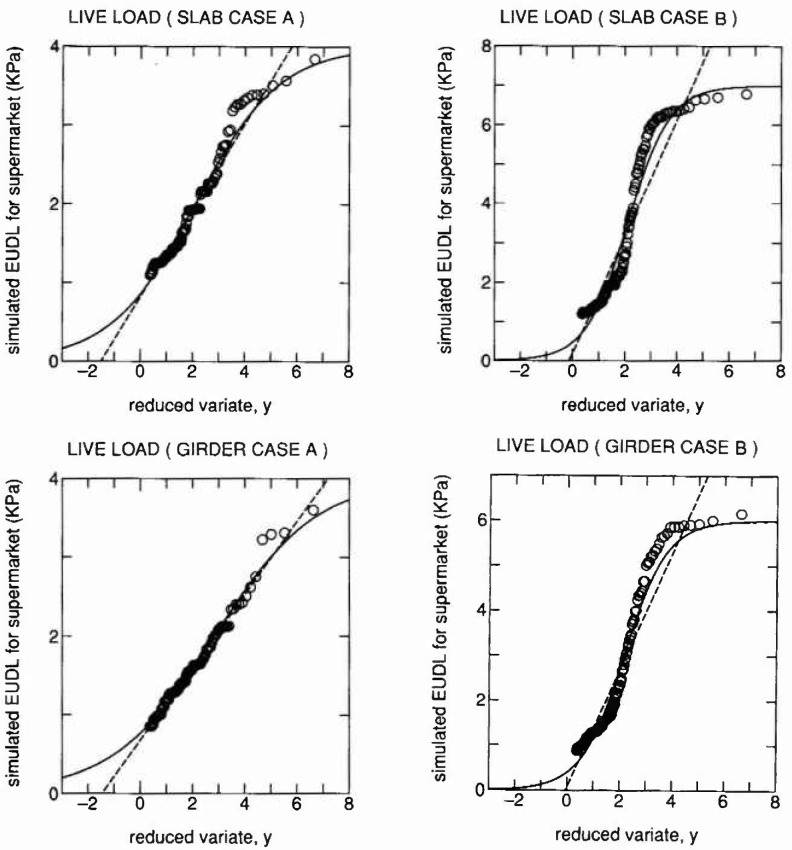
Extreme value fitting to simulated extraordinary live loads due to crowding concentration in supermarkets, where the solid and dashed lines indicate the proposed and the Gumbel distribution, respectively.

The plotted data with distribution curves of Eqs. (1) and (4) are shown in Fig. 5 in a similar manner to Figs. 3 and 4. Dashed lines represent the Gumbel distribution of Eq. (1). The upper half of the data were used to obtain parameters of distributions by the least squares method. The upper bound values of 4 kPa and 7 kPa were used for cases (a) and (b), respectively.

Results are summarized in Table 3. Saturation tendency is clear for case (b) where the error is significantly reduced by the proposed distribution. Assumed personnel load intensities in the scenario are somehow arbitrary and a further survey could improve models introduced herein. Nevertheless the usefulness of the proposed extreme value distribution with upper bound can be recognized.

## 6. Concluding Remarks

An empirical extreme value distribution with both upper and lower bounds was reviewed. The usefulness and improved fit to extreme load intensities available, such as the annual maximum earth* quake ground motion, the annual maximum wind speed and the extreme EUDL due to crowding situations in supermarkets, were demonstrated. Use of the simpler commonly used Gumbel or Frechet distributions could cause some significant overestimation in the coefficient of variation for lifetime maximum loads.

## Figures and Tables

**Table 1:** Statistics of maximum earthquake ground motion

		Proposed distribution, Eq (4)								Frechet distribution, Eq. (2)				
Site	*w*	*u*	*γ*	*E*	Annual max		50 year max		*c*	*γ*	*E*	Annual max		50 year max
		
					Mean	cov	Mean	cov				Mean	cov	Mean
Sendai	5.0	28.7	1.30	0.11	0.32	1.56	2.52	0.33	0.455	2.88	0.23	0.63	0.74	2.44
Tokyo	12.0	57.6	1.15	0.125	0.61	1.73	5.46	0.48	0.660	2.34	0.25	1.03	1.30	5.49
Osaka	10.0	90.9	1.18	0.21	0.34	1.99	3.48	0.60	0.303	2.06	0.37	0.52	3.29	3.50
Fukuoka	3.0	45.8	1.19	0.10	0.18	1.58	1.43	0.44	0.199	2.51	0.24	0.30	1.03	1.41

**Table 2:** Statistics of maximum wind speed

		Proposed distribution, Eq. (4)								Gumbel distribution, Eq. (1)					
Site	*w*	*u*	*γ*	*E*	Annual max		50 year max		*a*	*b*	*E*	Annual max		50 year max	
		
					Mean	cov	Mean	cov				Mean	cov	Mean	cov
Amori	35	1.09	3.00	0.02	18.4	0.19	27.9	0.08	0.37	16.8	0.02	18.4	0.19	29.1	0.12
Akita	35	0.70	2.98	0.02	22.0	0.14	30.1	0.05	0.41	20.6	0.03	22.0	0.14	31.7	0.10
Nagoya	40	1.63	2.79	0.08	17.3	0.25	29.8	0.10	0.29	15,4	0.08	17.4	0.25	30.9	0.14
Kagoshima	40	1.39	2.05	0.02	19.4	0.29	34.0	0.08	0.23	16.9	0.03	19.4	0.29	36.8	0.15

**Table 3:** Satistics of extreme EUDL for supermarkets

Case	Proposed distribution. Eq.(4)						Gumbel distribution Eq. (1)		
	*w*	*u*	*γ*	*E*	Mean	cov	*a*	*b*	*E*
Slab (a)	4000	3.65	1.61	0.04	1674	0.36	0.00182	827	0.07
Slab (b)	7000	13.6	0.80	0.11	2476	0.68	0.00067	193	0.24
Girder (a)	4000	4.22	1.96	0.03	1390	0.36	0.00216	676	0.04
Girder (b)	6000	14.3	0.81	0.10	2075	0.71	0.00076	64.7	0.22

## References

[1] Kanda J (1981). A New Extreme Value Distribution with Lower and Upper limits for Earthquake Motions and Wind Speeds. Theoretical and Applied Mechanics.

[2] Gumbel EJ (1958). Statistics of Extremes.

[3] Kawasumi H (1951). Measures of Earthquake Danger and Expectancy of Maximum Intensity throughout Japan as Inferred from the Seismic Activity in Historical Times. Bull Earthq Res Inst Tokyo.

[4] Kanda J, Dan K (1987). Distribution of Seismic Hazard in Japan based on an Empirical Extreme Value Distribution. Structural Safety.

[5] Usami T (1987). Revised List of Earthquakes Accompanied by Damages in Japan.

[6] Kanai K, Hirano K, Yoshizawa S (1966). Observation of Strong Earthquake Motions in Matsushiro Area, Part I. (Empirical Formulae of Strong Earthquake Motions). Bull Earthq Res Insl Tokyo.

[7] Kanda J, Saito T, Chung Y (1993). Return Period Estimation for Maximum Wind Speed caused by Typhoon 9119. Proc Struct Engr AIJ.

[8] Kanda J, Yamamura K (1989). Extraordinary Live Load Model in Retail Premises.

